# Phylogenetic analyses of *Lycium chinense* (Solanaceae) and its coordinal species applying chloroplast genome

**DOI:** 10.1080/23802359.2019.1669087

**Published:** 2020-06-01

**Authors:** Shui-Lian He, Yang Tian, Yang Yang, Chong-Ying Shi

**Affiliations:** a College of Horticulture and Landscape, Yunnan Agricultural University, Kunming, Yunnan, China; b Yunnan Key Laboratory of Biomass Big Data, Yunnan Agricultural University, Kunming, Yunnan, China; c College of Science, Yunnan Agricultural University, Kunming, Yunnan, China; d Institute of Food Science and Technology, Yunnan Agricultural University, Kunming, Yunnan, China

**Keywords:** *Lycium chinense*, medicinal and edible plant, chloroplast genome, phylogenetic analysis

## Abstract

*Lycium chinense* is an important edible and medicinal plant. Now, the complete chloroplast (cp) genome of *L. chinense* was assembled based on the Illumina sequencing reads. The cp genome of *L. chinense* was 155,736 bp long and contained two short inverted repeat regions (25,469 bp), which were separated by a small single-copy region (18,206 bp) and a large single-copy region (86,592 bp). The cp genome encodes 113 unique genes, including 79 protein-coding genes, 30 transfer RNA genes, and 4 ribosomal RNA genes. The topology of the phylogenetic tree showed that *L. chinense* is closely clustered with species *Lycium ruthenicum* and *Lycium barbarum*.

The genus *Lycium* contains several well-known medicinal and food plants in Solanaceae, such as *Lycium barbarum*, *Lycium chinense*, and *Lycium ruthenicum*. There are about 80 *Lycium* species around the world (Miller 2002; Levin and Miller 2005). *Lycium chinense* is food and herbal medicine in China and plays an important role in Chinese traditional medicine and has been used for thousands of years (Olivier 2009). The fruits of *L. chinense* have potential pharmacological effects such as anti-aging, reducing blood glucose and serum lipids, immune regulation, among others (Qin et al. 2001). In order to clarify the taxonomical position of *L. chinense* in Rosaceae, we applied the Illumina technology to sequence, assemble and annotate the whole chloroplast (cp) genome of *L. chinense*. The resultant data have been made publicly available as a resource for genetic information for *Lycium* species, and will provide a valuable plastid genomic resource for the future genetic and phylogenetic studies about *L. chinense.*

The fresh leaves of *L. chinense* were collected from the field of Kunming (25.20°N, 102.86°E). The voucher specimen was deposited at Herbarium of Yunnan Agricultural University (No. 2019HSL003). Total genomic DNA was isolated from fresh leaves using a DNeasy Plant Mini Kit (QIAGEN, Valencia, California, USA) according to the manufacturer’s instructions to construction cp DNA libraries. The Illumina sequencing was conducted by Biomarker Technologies Inc. (Beijing, China). Resultant clean reads were assembled using GetOrganelle pipeline (https://github.com/Kinggerm/GetOrganelle). The genome was automatically annotated by using the CpGAVAS pipeline (Liu et al. 2012) and start/stop codons and intron/exon boundaries were adjusted in Geneious R11.0.2 (Biomatters Ltd., Auckland, New Zealand). All the contigs were checked against the reference genome of *L. ruthenicum* (NC039651).

The complete cp genome of *L. chinense* was 159,898 bp in length (Genbank accession number: MN102357). It was the typical quadripartite structure and contained two short inverted repeat regions(IRa and IRb) of 26,540 bp each, which were separated by a small single-copy (SSC) region (19,219 bp) and a large single-copy (LSC) region (87,599 bp). The cp genome encodes 109 unique genes, including 75 protein-coding genes, 30 transfer RNA (tRNA) genes and 4 ribosomal RNA (rRNA) genes. Twenty gene species are partially or completely duplicated, including nine PCG (*ndhB*, *rpl2*, *rpsl23*, *rps12*, rps19, *rps7*, *ycf1*, *ycf2*, and *ycf15*), seven tRNA (*trnI-GAU*, *trnA-UGC*, *trnL-CAA*, *trnI-CAU*, *trnR-ACG*, *trnV-GAC*, and *trnN-GUU*), and all four rRNA (4.5S, 5S, 16S, and 23S rRNA). The overall GC content of the cp genome was 37.8%, while that of LSC, SSC, and IR regions was 35.9, 32.3, and 43.1%, respectively.

A total of 28 cp genome sequences were selected to infer the phylogenetic relationships among the main representative species of Solanaceae with *Magnolia alba* (*NC037005,* Magnoliaceae) as the outgroup. The combined datasets based on plastid genomes were aligned by MAFFT v7.307 (Katoh and Standley 2013). A neighbour-joining (NJ) phylogenetic tree was constructed in Geneious 11.1.5 (Kearse et al. 2012) with the Tamura–Nei genetic distance model, and a total of 1000 bootstrap replicates were performed. The topology of the phylogenetic tree showed that *L. chinense* has a close relationship with species *L. ruthenicum* and *L. barbarum* (Figure 1). The complete cp genome information reported in this study will be a valuable resource for future studies on genetic diversity, taxonomy, and phylogeny of the Solanaceae.

**Figure 1.**
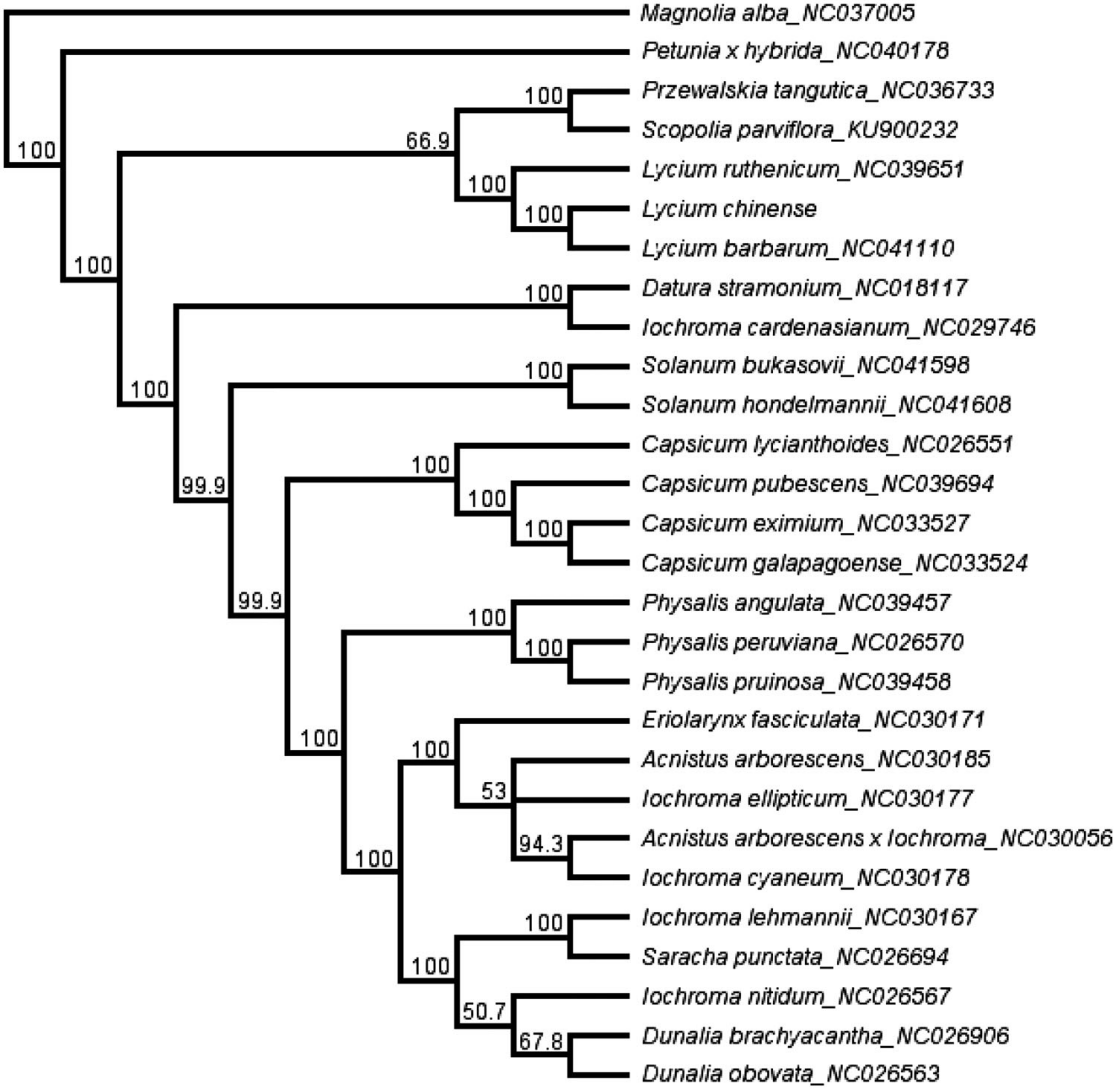
The neighbor-joining (NJ) phylogenetic tree based on 28 complete chloroplast genome sequences. Numbers at the right of nodes are bootstrap support values.
